# Coinfection with *Haemophilus parasuis serovar* 4 increases the virulence of *porcine circovirus* type 2 in piglets

**DOI:** 10.1186/s12985-017-0890-6

**Published:** 2017-11-21

**Authors:** Shuqing Liu, Wentao Li, Yang Wang, Changqin Gu, Xiaoli Liu, Catherine Charreyre, Shenxian Fan, Qigai He

**Affiliations:** 1 State Key Laboratory of Agricultural Microbiology, College of Animal Science and Veterinary Medicine, Huazhong Agricultural University, Wuhan, Hubei 430070 China; 2 Key Laboratory of Medical Virology, Ministry of Health, National Institute for Viral Disease Control and Prevention, Chinese Center for Disease Control and Prevention, Beijing, 102206 China; 3 Merial S.A.S, 254 rue Marcel Mérieux, 69007 Lyon, France; 4 Department of Animal Infectious Disease, State Key Laboratory of Agricultural Microbiology, Huazhong Agricultural University, Wuhan, Hubei 430070 China

**Keywords:** PMWS, PCV2, HPS4, Coinfection, NFCD piglets

## Abstract

**Background:**

Postweaning multisystemic wasting syndrome (PMWS) is an emerging disease in swine. Pigs with PMWS are often infected with a variety of other pathogens, including bacteria, viruses and mycoplasm, in addition to porcine circovirus type 2 (PCV2). PCV2 and Haemophilus parasuis serovar 4 (HPS4) coinfection remain epidemic in China.

**Methods:**

Here we report construction of a three-week-old naturally farrowed, colostrum-deprived (NFCD) piglet’s infection model and demonstrate that PCV2-infected piglets with the HPS4 coinfection increased the virulence of PCV2 and these pathogens interact acquired PMWS.

**Results:**

All the single infected piglets were transiently bacteremic or viremic. All the PCV2/HPS4 coinfected piglets developed PMWS, characterized by dyspnea, anorexia, prostration and lose weight severely. Co-infection with PCV2 and HPS4 resulted in an increased amount of virus in serum and tissues, presented a slower generation and lower levels of antibodies against PCV2. Co-infection with PCV2 and HPS4 resulted in further reductions in total and differential peripheral blood leukocyte counts. Meantime, PCV2/ HPS4 coinfection potentiated the severity of lung and lymphoid lesions by PCV2-associated, increased the virulence of PCV2-antigen and enhanced the incidence of PMWS in piglets.

**Conclusion:**

Co-infection with PCV2 and HPS4 induce the exacerbation of system injuries and enhance the pathogenicity of PCV2 in piglets.

## Background

Porcine circovirus type 2 (PCV2) has been identified as the causal agent of postweaning multisystemic wasting syndrome (PMWS), an economically important multifactorial disease of swine industry worldwide [1, 2]. PMWS is characterized by weight loss, jaundice, generalized lymphadenopathia, interstitial pneumonia and nephritis [3]. Based on the broad clinical and serological investigations, Multiple factors have contributed the PCV2 infections as PMWS, such as porcine reproductive and respiratory syndrome virus(PRRSV), porcine parvovirus (PPV), Haemophilus parasuis(HPS), and Actinobacillus pleuropneumoniae (APP), a variety of viral and bacterial pathogens in the majority of cases (85.0%) [4, 5].

HPS is an important swine pathogen that causes Glässer’s disease, which is characterized by fibrinous polyserositis, meningitis and arthritis [6]. Clinically, HPS co-infection with other pathogens, such as PRRSV, PCV2, *Streptococcus suis* (SS) and *Pasteurella multocida* (Pm), lead to increasing economic losses in the swine industry worldwide [5–8]. Previous observations indicated that PCV2 and HPS exacerbate secondary or opportunistic infections, co-infection with PCV2 and HPS was the most prevalent combination associated with PMWS in Korea and China, HPS serovar 4 (24.2%) and HPS serovar 5 (19.2%) were the most prevalent serovars in China [5, 8, 9]. Therefore, We established a porcine model to elucidate the clinical changes of PCV2 and HPS4 co-infection, using clinical isolates of PCV2 genotype 2b WH strain and HPS4 MD0322 strain isolated from china, based on the protocol for coinfection model establishment proposed by Harms et al. [10]. We firstly measured the clinical and peripheral blood changes of PCV2 and HPS4 co-infection, in order to analyze the synergistic influence on the virulence of PCV2 in piglets.

## Methods

### Experimental design and inoculations

Three-week-old naturally farrowed, colostrum-deprived (NFCD) piglets were used, obtained from a herd in Hubei province without PCV2, PPV, PRRSV, SS2 or HPS pathogen. All piglets were randomly divided into four groups (six piglets per group) and raised separately in four isolation rooms with individual ventilation. Animals received food and water ad libitum. After one week, all piglets were inoculated and slaughtered at 28 day post infection (DPI). The Clinical separated strain PCV2 genotype 2b (WH) was used as the source of viral inoculums (GenBank ID:FJ870967). The titer of the virus was calculated as 10^7.3^ TCID_50_/ml. HPS4 strain MD0322 was used as the source of bacterial inoculums. It was isolated from the Zhijiang city of Hubei province emergency on August 2001 as the cases of fibrinous, polyserositis and arthritis in piggery. The bacterial inoculum contained approximately 4.5 × 10^8^ colony- forming units (CFU)/ml. The piglets in the PCV2-infected group and PCV2/HPS4 coinfected group were intramuscularly (IM; 2.5 ml) and intranasally (IN; 2.5 ml) inoculated with the WH strain of PCV2. The pigs in the HPS4-infected group and PCV2/HPS4 coinfected group were IM (2.5 ml) and IN (2.5 ml) inoculated with the HPS4 strain MD0322. The piglets in the control group were inoculated with DMEM in the same way. All animal experiments were complied with the requirement of animal welfare organizations and approved by the Biological Studies Animal Care and Use Committee of the Hubei Province, People’s Republic of China.

### Clinical evaluation

After PCV2 and HPS4 challenge, piglets were monitored daily and scored for clinical signs including rectal temperatures, prostration, dyspnea, cough, anorexia, limping vomiting and other symptoms: MO mortality, TR trembling, CY cyanosis, DI diarrhea. The piglets were observed and weighed from 3 days prior-challenge to 28 days post-challenge, finally slaughtered at 28 DPI. Clinical Observation Record is summarized in Table 1.

**Table 1:** Clinical Observation Record

Group	Prostration	Dyspnea	Caugh	Anorexia	Limping	Vomiting	Other symptoms
Control	0/6	0/6	0/6	0/6	0/6	0/6	None
PCV2	4/6	3/6	0/6	2/6	0/6	0/6	TM, DR
HPS4	4/6	2/6	3/6	3/6	5/6	0/6	TM
PCV2/HPS4	5/6	3/6	3/6	3/6	6/6	0/6	TM, DR

All piglets will be observed for signs of prostration, dyspnea, cough, anorexia, limping and vomiting from 3 days prior-challenge to 28 days post-challenge.*Other symptoms: *MO* mortality, *TM* trembling, *LM* lame, *DR* diarrhea, *ND* Not Done

### Detection of antibodies to PCV2 by indirect ELISA

Blood samples were collected on 0, 1, 3, 5, 7, 14, 21 and 28 DPI. Anti -PCV2 ORF2 antibodies were tested by enzyme-linked immunosorbent assays (ELISA) as Chun described previously [11]. The calibrated OD for each tested and control serum was calculated by subtraction of mean OD of the wells containing negative antigen from that of the parallel wells containing PCV2 antigen. The data were normalized by dividing the calibrated OD of a tested serum sample by that of the positive control serum and were reported as the sample/positive (S/P) ratios. The samples with serum sample/positive control serum ratios of ≤0.2 and >0.2 were considered negative and positive, respectively. In addition, the calibrated OD for the positive serum control had to be equal or higher than 0.4 for the assay to be valid.

### Quantification of PCV2 genomes by real-time PCR in tissues

Tissues were grinded into homogenate supernatants and Virus DNA was extracted with Viral DNA Kit (OMEGA, USA). Viral loads in tissues were quantificated by a TaqMan real-time PCR [12]. The reactions were performed on an ABI Prism 7500 thermocycler (Applied Biosystem, Foster City, CA, USA).

### Total white blood cell (WBC) counts

Total WBC counts in EDTA-stabilized blood were measured using Beckman-Coulter Ac.T diff 2 automated haematological analyzer (Coulter Corp, A Beckman Coulter Co, Miami, Florida).

### Histopathology

All piglets were inoculated and slaughtered at 28 DPI. Samples of lung, lymph nodes, tonsil, kidney, spleen, heart, liver and brain were collected and fixed in 10% neutral buffered formalin for 2–4 days, then embedded in paraffin for hematoxylin and eosin (HE) straining. Histopathology examination was observed with optical microscope (Olympus).

### Statistical analysis

The data were analyzed using independent sample T-test with the computing software Statistical Package for the Social Sciences (SPSS), and the results were expressed as mean value ± standard error of the mean (S.E.M.). A *P*-value of results was considered significant at probability (*P*-values ≤0.05).

## Results

### Clinical evaluation

The piglets in PCV2-infected and PCV2/HPS4 coinfected group had fever (rectal temperatures >40 °C) and severe emaciation than control and HPS4-infected group. The average daily gain of PCV2-infected group (263.10 ± 41.01 g/day), PCV2/HPS4 coinfected group (227.38 ± 42.96 g/day) or HPS4-infected group (333.33 ± 90.02 g/day) was statistically significant (*p* < 0.05) for control group (437.05 ± 21.35 g/day) respectively. PCV2-infected piglets exhibited prostration (4/6), dyspnea (3/6) and anorexia (2/6), 2 piglets appeared severe trembling and diarrhea at 8–14 DPI. The HPS4 infected piglets showed mild trembling (2/6), limping (5/6) and palpebra hyperemia (4/6). Among the PCV2/HPS4 coinfected piglets by clinical observation, 5 piglets exhibited prostration, pyrexia, lymphadenectasis and bloody ocular secretions at 3 DPI, and 3 piglets showed severe cough, dyspnea, anorexia and diarrhea at 6 DPI. All piglets exhibited varying degrees of joint swelling or limping, and 2 piglets led to posterior limb paralysis finally. The relevant clinical data are shown in Table 1.

### Co-infection with PCV2 and HPS4 resulted in an increased amount of virus in serum and tissues

**Fig. 1:**
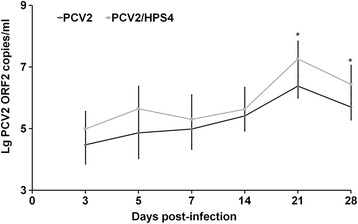
Serum PCV2 viral loads in the infected piglets. The amounts of PCV2 in serum were determined by real-time PCR and expressed as the mean logarithm of PCV2 ORF2 DNA copy numbers per milliliter (*n* = 6 in each group). Error bars represent standard deviations. *p < 0.05

**Fig. 2:**
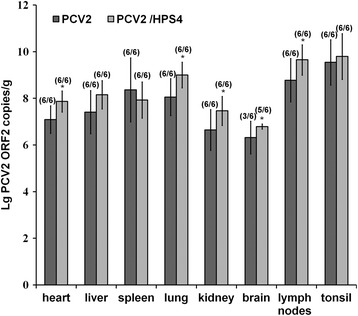
PCV2 viral loads in tissues in the infected piglets. The amounts of PCV2 in tissues were determined by real-time PCR and expressed as the mean logarithm of PCV2 ORF2 DNA copy numbers per gram (n = 6 in each group). Error bars represent standard deviations. *p < 0.05

No PCV2 antigen was detected in control or HPS4-infected group by Real-time PCR in the whole experiment. The kinetics of serum viral loads was shown in Fig. 1. PCV2-infected or PCV2/HPS4 coinfected group exhibited a dramatic increase in PCV2 serum load from 3 DPI. Meanwhile, the PCV2 viral loads in PCV2/HPS4 coinfected piglets were significantly higher than in PCV2-infected piglets at 21 DPI and 28 DPI, the maximum level of which in PCV2/HPS4 coinfected piglets reached to 1.87 × 10^8^ copies/ml at 21 DPI. The PCV2 genome loads in tissues are summarized in Fig. 2. All piglets in PCV2-infected and PCV2/HPS4 coinfected groups contained PCV2 antigen in the heart, liver, spleen, lung, kidney, lymph nodes and tonsil. 5 piglets in coinfected group and 3 piglets in PCV2-infected group contained PCV2 antigen in the brain. The maximum value of PCV2 genome loads reached to 2.04 × 10^10^ copies/g in PCV2-infected (2.04 × 10^10^ copies/g) and 3.17× 10^10^ copies/g in PCV2/HPS4 coinfected piglets from the tonsil. The PCV2 loads of heart, lung, kidney, brain and lymph nodes in PCV2/HPS4 coinfected piglets were significantly higher (*p* < 0.05) than that in PCV2-infected piglets.

### Co-infection with PCV2 and HPS4 resulted in slower generation and lower levels of antibodies against PCV2

**Fig. 3:**
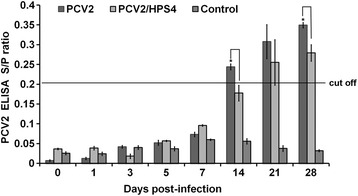
Detection of anti-PCV2 antibodies. Serum samples were collected on days 0, 1, 3, 5, 7, 14, 21 and 28, respectively, and anti-PCV2 antibodies determined by ELISA. The horizontal line represents cut off value for assay

Indirect ELISA was performed to detect antibodies against PCV2. The mean PCV2 antibodies S/P (sample/positive) ratios of pig serum collected were determined (Fig. 3). All piglets were negative for antibodies against PCV2 before inoculation. The control and HPS4-infected group remained seronegative to PCV2 throughout the experiment. PCV2-specific antibodies were seroconverted at 14 DPI in PCV2 group, and in PCV2/HPS4 coinfected group, antibodies to PCV2 were detected for the first time at 14 DPI in 3 piglets, and at 21 DPI in the other 3 piglets. The levels of the PCV2 antibody were lower in PCV2/HPS4 coinfected group than that in PCV2-infected group during the whole experimental period and were significantly lower at 14 and 28 DPI.

### Co-infection with PCV2 and HPS4 resulted in further reductions in total and differential peripheral blood leukocyte counts

**Fig. 4:**
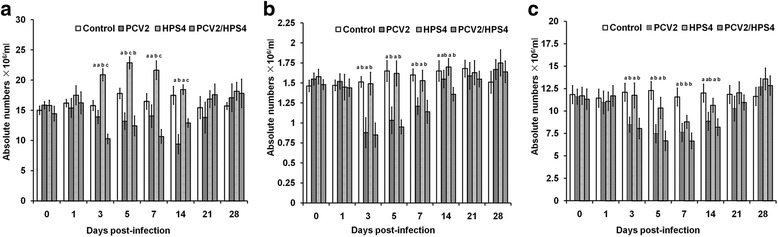
Changes of total and differential leukocyte counts in the peripheral blood of the infected piglets. (1) Total white blood cells (WBC); (2) monocytes; (3) lymphocytes. The values represent the mean of the absolute numbers (n = 6 in each group); error bars show standard deviations. Data with different letters (**a**-**c**) indicate significant differences between groups (p < 0.05)

The total number of WBC in PCV2/HPS4 coinfected group was significantly decreased at 3, 7 and 14 DPI in comparison to the other three groups (Fig. 4a), and the total WBC Count in PCV2-infected group was significantly lower than the other three groups at 14 DPI. However, the WBC counts in HPS4-infected group appeared significantly higher than the other three groups from 3 DPI to 7 DPI. The total number of monocytes in PCV2/HPS4 coinfected group was significantly lower at 3, 5, 7 and 14 DPI compared to the control and HPS4-infected group (Fig. 4b), while the number of monocytes in PCV2-infected group decreased significantly at 3, 5 and 7 DPI in comparison to the control group. The number of peripheral blood lymphocytes in PCV2/HPS4 coinfected group was significantly reduced at 3, 5 and 14 DPI in comparison to the control and HPS4-infected group (Fig. 4c). Compared with the control group, the total number of lymphocytes in PCV2-infected group decreased significantly at 3, 5 and 7 DPI, while the number of lymphocytes in the HPS4-infected group was significantly lower at 7 DPI. The number of granulocytes in HPS4-infected group significantly increased at 5 and 7 DPI in comparison to the control group (data not shown).

### Co-infection with PCV2 and HPS4 resulted in severe microscopic lesions

**Fig. 5:**
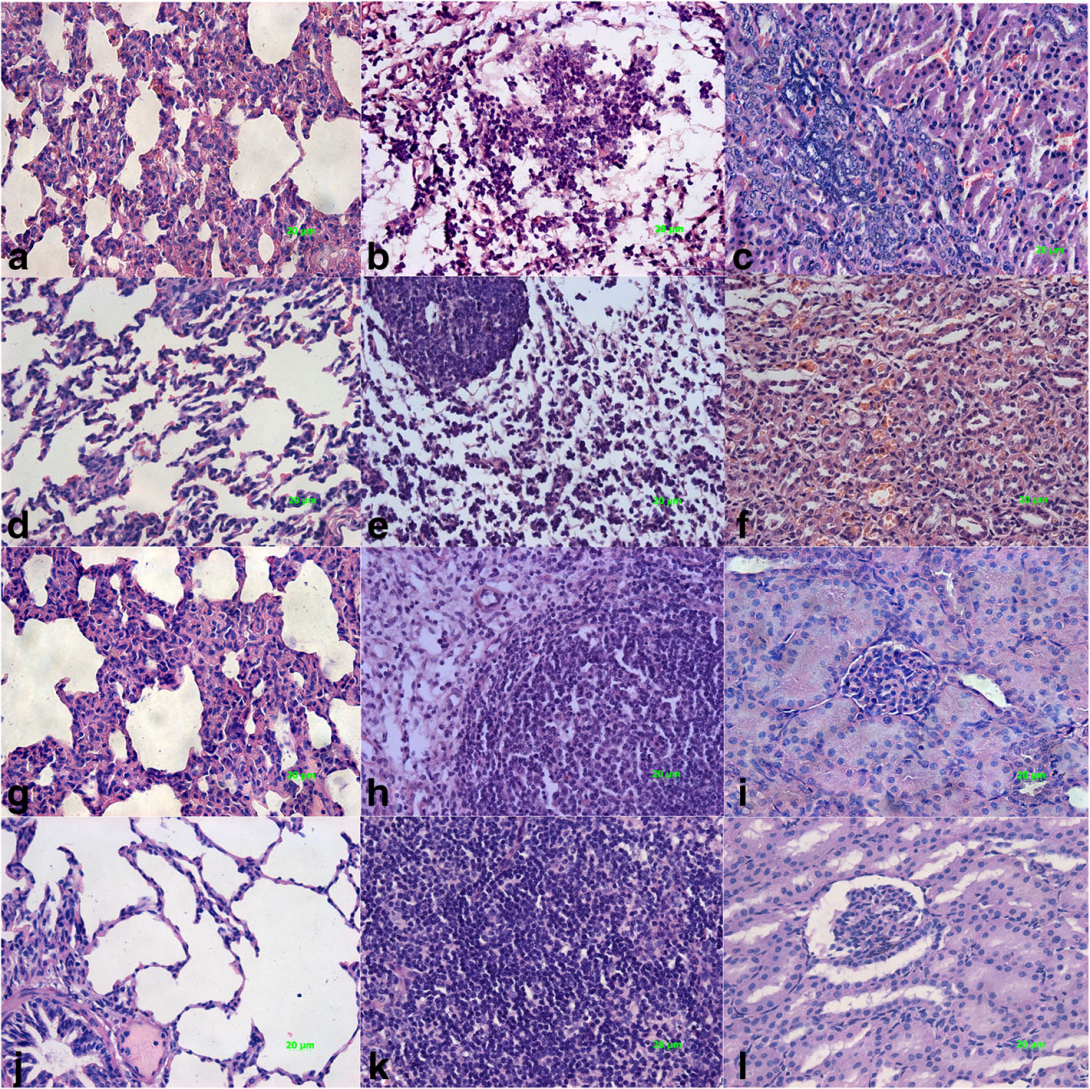
Microscopic lesions in four groups. Note: **a**, **b**, C respectively represents pathological characters of lung, lymph node and kidney in PCV2/HPS4-coinfected group, severe pneumonia with widened alveolar septa in lung, lymphocyte depletion and disintegration in lymph node, mild focal hyperplasia of the mesenchymal cells in kidney. **d**, **e**, **f** respectively represents pathological characters of lung, lymph node and kidney in PCV2-infected group. **g**, **h**, l respectively represents pathological characters of lung, lymph node and kidney in HPS4-infected group; **j**, **k**. **l** respectively represents pathological characters of lung, lymph node and kidney in control group. The scale = 20um, HE

The pathological lung lesions in PCV2/HPS4-coinfected piglets showed severe widened alveolar septa and lymphocytes infiltration (Fig 5a). The lymphoid nodes lesions showed severe lymphocyte depletion and disintegration (Fig 5b). The kidney lesions in PCV2/HPS4-coinfected piglets included mild focal hyperplasia of the mesenchymal cells (Fig 5c). The histopathologic lung lesion in PCV2-infected group mainly exhibited a small amout of lymphocytes infiltration (Fig 5d). The lymphoid nodes lesions showed a considerable degree of depletion, and germinal center atrophy in lymph nodule (Fig 5e). The kidney lesions in showed renal vein expansion and congestion (Fig 5f). The lungs lesions in HPS4-infected group followed by lymphocytes infiltration and widened alveolar septa (Fig 5g). No obvious lymphoid nodes lesions were observed (Fig 5h), and the kidney lesions were swelling in epithelial cells of proximal tubule (Fig 5i). No pathological lesions were observed in control group tissues (Fig. 5j-l).

## Discussion

PMWS is one of the most significant porcine diseases worldwide, and the causative agent is porcine circovirus type 2 (PCV2) [1–3].Several reports have confirmed that, the coinfection of PCV2 and HPS are pervasive in piglets, it has been a great threat in the pig industries globally [5, 7, 8]. The isolation rate of serovar 4 was most prevalent in China, similar results were also reported in North America [8, 13, 14]. In this study, we found that PCV2 alone cannot cause extensive damage to the host. The piglets coinfected with PCV2(WH) and HPS4 (MD0322) showed symptoms similar to PMWS: high fever, severe emaciation and lymphadenectasis. The HPS4 strain MD0322, an isolate obtained from a PMWS endemic area, was weakly pathogenic in piglets [15]. In this study, the clinical symptoms of the HPS4-infected piglets were mild, because the self-healing capabilities of piglets may start within a short duration. However, the condition of piglets coinfected with PCV2 and HPS4 deteriorated rapidly, the rectal temperature of all piglets reached 41.5 °C at 7 DPI, and the symptoms of PMWS, such as severe weight loss and lymphadenectasis appeared at 3 DPI. Therefore, we suggest that even HPS4, which is a weak virulent serotype, can cause more damage significantly when coinfected with PCV2. This is the first report that PCV2-infected piglets with the HPS4 coinfection increased the virulence of PCV2 and the incidence of PMWS in experimental NFCD piglet’s model.

In this study, Our data demonstrated that PCV2/HPS4 co-infected piglets had higher viral loads and a longer viremia duration in comparison with PCV2 single infection. Meanwhile, co-infection with PCV2 and HPS4 resulted in an increased amount of PCV2 genome loads in heart, liver, spleen, lung, kidney, lymph nodes, especially from the tonsil. Bacterial lipopolysaccharide (LPS) induced PCV2 replication in swine alveolar macrophages [16], hence, HPS4 may enhance the PCV2 replication at the site of infection, finally increases the amount of PCV2 viral loads in lymphoid tissue and immune organs significantly. This suggests that co-infection with PCV2 and HPS4 induce the exacerbation of system injuries and aggravates the secondary bacterial infection.

Since the monocyte/macrophage cell lines are common targets of PCV2 virus [17], co-infection can result in immunomodulation and enhanced the replication of PCV2. The previous study showed a dramatic reduction in the number of B lymphocytes with PCV2 infection [11], and then B cells elimination may result in PCV2 antibody production limit and immunosuppression. That may explain the PCV2/HPS4 co-infected piglets showed a slower generation and lower amounts of PCV2-specific antibodies than that in piglets with PCV2 infection alone. This suggests that coinfection exacerbates depression of the host’s immune responses. So we measured the changes in total WBC, monocytes, lymphocytes and granulocytes to study the effects of PCV2 and HPS4 coinfection on the innate immune response of piglets. The total number of WBC, monocytes and lymphocytes in PCV2/HPS4 coinfected group was significantly decreased for about two weeks during the early stage of infection, which is consistent with the previous reports [18]. Lymphocyte subpopulations are crucial elements of the immune system and pivotal for the control and elimination of viruses [19]. The dramatic reduction of lymphocytes following infection reflected immunosuppression in the in PCV2/HPS4 coinfected piglets. Demonstration of viral DNA in blood with the concurrent serum antibody response to PCV-2 suggested that PCV-2 infection may be persistent. Persistent DNA viremia after onset of specific antibody indicates that developing humoral antibodies were ineffective in virus clearance after infection [20]. Thus, PCV2 and HPS4 co-infection exhibited a greater reduction in immune effector cells, inhibited and exacerbated the depression the host defense more severely than either PCV2 or HPS4 infection alone, although produced lower antibodies response to PCV-2 which was ineffective in PCV2 virus clearance.

Several reports have confirmed that the main characteristic of PCV2 is the damaging effects on the host immune system, including interstitial pneumonia and lymphoid depletion, which facilitates the invasion of other pathogens and leads to the cases of PMWS [19]. In this study, the lung and lymphoid tissues lesions showed severe widened alveolar septa and lymphocyte depletion than singly infected piglets. Segales et al. believed that PCV2 infection had compromised the effectiveness of host’s immune response against lungworms [21], thus triggering a diffuse, severe and fatal parasitic bronchopneumonia. The higher viral load in the lymph nodes likely reflected the fact that lymphoid organs are severely affected in PMWS animals [22]. This result was consistent with the findings of previous reports the distribution of antigen and PCV-2 DNA in different tissues indicated that the lymphoid tissues of pigs with PMWS were the main sites of replication of PCV-2 [17]. In addition, it is known that PCV-2 could infect dividing cells, macrophages and B lymphocytes, inducing apoptosis in B cells and causing lymphoid depletion [23]. Lymphoid depletion was observed in most of the lymph nodes that were analyzed in PCV2/HPS4-coinfected group. The frequency of occurrence of microscopical lesions in the kidney was related to the viral load, and this finding can be explained by the fact that the kidney is known to be a site of immune complex deposition in animals with PCV-2 infection [22, 24]. PCV-2 induces the dramatic reduction of lymphocytes following PCV2 infection reflects immunosuppression [25], which may make pigs more susceptible to HPS infection, thereby secondary infection by HPS4 improved the distribution of the PCV2, further exacerbating the damage to the immune system and the pathological lesions to the lung, lymphoid tissues and kidney. The combined effect of PCV2 and HPS4 coinfection increased the host injuries significantly. Therefore, this study demonstrates that coinfection with HPS4 enhance the pathogenicity of PCV2 in piglets.

It has been demonstrated that dramatic reduction of lymphocytes following PCV2 infection reflects immunosuppression in the infected piglets [25], and PCV2 replication is enhanced by coinfection with viruses such as porcine parvovirus (PPV) [26, 27], PRRSV [10, 28], porcine epidemic diarrhoea virus (PEDV) [29] and porcine torque tenue virus(TTV) [30]. Marion et al. considered PCV2 infection maked animals more susceptible to co-infections with PRRSV and *Mycoplasma hyopneumoniae* (Mhp), and vaccination against PCV2 alone could lower the incidence of coinfecting agents such as PRRSV and *Mycoplasma hyorhinis* (Mhr) in PMWS-affected animals [31–33]. There is also evidence suggesting that the commonality of porcine immune function target cells may play a key role in this synergism for these two viruses due to stimulation of the monocyte/macrophage lineage and other cells of the immune system [34, 35]. Opriessnig et al. reported that Mhp prior infection could up-regulating the macrophage proliferation and create an ideal environment for PCV2 replication [36]. No matter the co-infection times of the two pathogens, Mhp might enhance PCV2 replication for multiple reasons [37]. PCV2 also could be enhanced by indirect initiation of host cell replication by other pathogens. The cell cycle could be initiated by other pathogens if they infect the same cell as PCV2. Opriessnig et al. also found that PCV2 and *S. typhimurium* antigens were occasionally detected within the same macrophage-like cells [38]. LPS is the main endotoxin of Gram-negative bacteria and released at high concentrations in the lungs during pulmonary infection with Gram-negative bacteria [39, 40]. Chang et al. found in PK-15 cells for both PCV2 antigens and nucleic acid were successfully demonstrated in LPS-treated PCV2-inoculated alveolar macrophages [16]. Thus, we speculated that PCV-2 induced the dramatic reduction of lymphocytes following PCV2 infection reflects immunosuppression, which maked pigs more susceptible to HPS infection, according to the stimulation of the monocyte/macrophage lineage and other cells of the immune system by HPS surface component LPS, PCV2 could be initiated to replicate finally.

A positively synergistic interaction effect on the mRNA expression of CD14, TLR4 and TLR8 might enhance the production of pro-inflammatory cytokines such as IL-1β [41]. Experimental exposure of pigs to LPS following porcine respiratory coronavirus (PRCV) infection has led to more severe respiratory disease and increased TNF-α and IL-1β levels [42]. PRRSV infection often made the host more susceptibility to *Haemophilus parasuis* (HPS). The combination of PRRSV HN07–1 strain and LPS exhibits significant synergistic effects on the secretion of TNF-α and IL-1β produced by macrophages in response and in the induction of an increase CD14 expression in the porcine alveolar macrophages (PAMs) [43, 44]. The simultaneously and highly enhanced expressions of TLR4 and CD14 in PBMCs of pigs co-infected with PRRSV and PCV2 might render the animal vulnerable to secondary bacterial infection observed in the field [45]. Fu et al. reported HPS can initiate innate immune responses and induce the production of inflammatory cytokines [46]. HPS RNA could significantly enhanced HP-PRRSV infection-mediated inflammatory responses in PAMs to promote the enhancement of NLRP3 inflammasome activation and IL-1β secretion [47]. Thus, we speculated that HPS enhanced PCV2 infection-mediated inflammatory responses, which effected on the mRNA expression of TLR4 and enhance the production of inflammatory cytokines TNF-α and IL-1β secretion by bacterial LPS stimulation and HPS RNA, which could induce the PCV2 replication.

In addition, the 70 kDa heat-shock proteins (Hsp70) is one of the most important heat shock proteins, it has been proposed as one of the candidate vaccine antigens of Mhp [48, 49], we have found the Hsp70 was up-regulated in microarray also showed significantly higher expression in *H. parasuis* serovar 5 infected samples than in the control samples [50]. Evidence is growing that HSP70 plays important roles in the replication of many viruses, and it enhanced PCV2 genome replication and virion production via the interaction with PCV2 cap in vitro cells [49, 51, 52]. Thus we speculated that Hsp70 of HPS4 might be one of the factors that enhanced PCV2 replication. Taken together, we speculated that PCV-2 induced the dramatic reduction of lymphocytes following PCV2 infection reflects immunosuppression, which maked pigs more susceptible to HPS infection. The stimulation of the monocyte/macrophage lineage and immune response by surface component LPS, RNA and Hsp70 of HPS4, contributed to induce the PCV2 replication, thereby improved the distribution of the PCV2, further exacerbating the damage to the immune system and potentiating the severity of PMWS in pigs.

## Conclusions

We have shown that the clinical manifestations of the coinfection model are similar to those observed during the PMWS epidemic in China. The HPS4 epidemic may have played a role in the occurrence and development of PMWS. The general level of importance of HPS4 has always been less than HPS5 because of the characteristic low virulence of the former. However, piglets coinfected with PCV2 and HPS4 exhibited serious clinical symptoms affecting multiple organs, especially the lungs, lymph nodes and kidney. Therefore, it is necessary to control bacterial infection by adopting measure to prevent and control PCV2 infection.

## Abbreviations

APP: Actinobacillus pleuropneumoniae
CY: cyanosis
DI: diarrhea
DPI: day post infection
ELISA: enzyme-linked immunosorbent assays
HPS4: Haemophilus parasuis serovar 4
IM: intramuscularly
IN: intranasally
Mhp: Mycoplasma hyopneumoniae
Mhr: Mycoplasma hyorhinis
MO: mortality
NFCD: naturally farrowed, colostrum-deprived
PAMs: porcine alveolar macrophages
PCV2: Porcine circovirus type 2
PEDV: porcine epidemic diarrhoea virus
Pm: *Pasteurella multocida*
PMWS: postweaning multisystemic wasting syndrome
PPV: porcine parvovirus
PRRSV: porcine reproductive and respiratory syndrome virus
SS: *Streptococcus suis*
TR: trembling
TTV: porcine torque tenue virus

## References

[1] Bolin SR, Stoffregen WC, Nayar GP (2001). Postweaning multisystemic wasting syndrome induced after experimental inoculation of cesarean-derived, colostrum-deprived piglets with type 2 porcine circovirus. J Vet Diagn Investig.

[2] Ellis JA, Bratanich A, Clark EG (2000). Coinfection by porcine circoviruses and porcine parvovirus in pigs with naturally acquired postweaning multisystemic wasting syndrome. J Vet Diagn Investig.

[3] Allan GM, Ellis JA (2000). Porcine circoviruses: a review. J Vet Diagn Investig.

[4] Choi C, Chae C (2001). Colocalization of porcine reproductive and respiratory syndrome virus and porcine circovirus 2 in porcine dermatitis and nephropathy syndrome by double-labeling technique. Vet Pathol.

[5] Kim J, Chung HK, Jung T (2002). Postweaning multisystemic wasting syndrome of pigs in Korea: prevalence, microscopic lesions and coexisting microorganisms. J Vet Med Sci.

[6] Ru’bies X, Kielstein P, Costs LI (1999). Prevalence of Haemophilus parasuis serovars isolated in Spain from 1993 to 1997. Vet Microbiol.

[7] Solano GI, Segales J, Collins JE (1997). Porcine reproductive and respiratory syndrome virus (PRRSv) interaction with Haemophilus parasuis. Vet Microbiol.

[8] Cai X, Chen H, Blackall PJ (2005). Serological characterization of Haemophilus parasuis isolates from China. Vet Microbiol.

[9] Li JX, Jiang P, Wang Y (2009). Genotyping of Haemophilus parasuis from diseased pigs in China and prevalence of two coexisting virus pathogens. Prev Vet Med.

[10] Harms PA, Sorden SD, Halbur PG (2001). Experimental reproduction of severe disease in CD/CD pigs concurrently infected with type 2 porcine circovirus and porcine reproductive and respiratory syndrome virus. Vet Pathol.

[11] Ju CM, Fan HY, Tan YD (2005). Immunogenicity of a recombinant pseudorabies virus expressing ORF1-ORF2 fusion protein of porcine circovirus type 2. Vet Microbiol.

[12] Olvera A, Sibila M, Calsamiglia M (2004). Comparison of porcine circovirus type 2 load in serum quantified by a real time PCR in postweaning multisystemic wasting syndrome and porcine dermatitis and nephropathy syndrome naturally affected pigs. J Virol Methods.

[13] Oliveira S, Blackall PJ, Pijoan C (2003). Characterization of the diversity of Haemophilus parasuis field isolates by serotyping and genotyping. Am J Vet Res.

[14] Tadjine M, Mittal KR, Bourdon S (2004). Development of a new serological test for serotyping Haemophilus parasuis isolates and determination of their prevalence in North America. J Clin Microbiol.

[15] Yuan F, Fu S, Hu J (2012). Evaluation of recombinant proteins of Haemophilus parasuis strain SH0165 asvaccine candidates in a mouse model. Res Vet Sci.

[16] Chang HW, Pang VF, Chen LJ (2006). Bacterial lipopolysaccharide induces porcine circovirus type 2 replication in swine alveolar macrophages. Vet Microbiol.

[17] Rosell C, Segales J, Plana-Duran J (1999). Pathological, immunohistochemical, and in-situ hybridization studies of natural cases of postweaning multisystemic wasting syndrome (PMWS) in pigs. J Comp Pathol.

[18] Nielsen J, Vincent IE, Botner A (2003). Association of lymphopenia with porcine circovirus type 2 induced postweaning multisystemic wasting syndrome (PMWS). Vet Immunol Immunop.

[19] Shi KC, Li HR, Guo X (2008). Changes in peripheral blood leukocyte subpopulations in piglets co-infected experimentally with porcine reproductive and respiratory syndrome virus and porcine circovirus type 2. Vet Microbiol.

[20] Krakowka S, Ellis JA, Meehan B (2000). Viral wasting syndrome of swine: experimental reproduction of Postweaning multisystemic wasting syndrome in Gnotobiotic swine by Coinfection with porcine Circovirus 2 and porcine parvovirus. Vet Pathol.

[21] Segales J, Rosell C, Domingo M (2004). Pathological findings associated with naturally acquired porcine circovirus type 2 associated disease. Vet Microbiol.

[22] Yu S, Opriessnig T, Kitikoon P (2007). Porcine circovirus type 2 (PCV2) distribution and replication in tissues and immune cells in early infected pigs. Vet Immunol Immunopathol.

[23] ShibaharaT SK, Ishikawa Y (2000). Porcine circovirus induces B lymphocyte depletion in pigs with wasting disease syndrome. J Vet Med Sci.

[24] Segalés J, Domingo M, Chianini F (2004). Immunosuppression in postweaning multisystemic wasting syndrome affected pigs. Vet Microbiol.

[25] Opriessnig T, Langohr I (2013). Current state of knowledge on porcine circovirus type 2-associated lesions. Vet Pathol.

[26] Allan GM, Kennedy S, McNeilly F, et al. Experimental reproduction of severe wasting disease by co-infection of pigs with porcine circovirus and porcine parvovirus. J Comp Pathol 1999:1:1-11. 10.1053/jcpa.1998.0295 10373289

[27] Opriessnig T, Fenaux M, Yu S (2004). Effect of porcine parvovirus vaccination on the development of PMWS in segregated early weaned pigs coinfected with type 2 porcine circovirus and porcine parvovirus. Vet Microbiol.

[28] Allan GM, McNeilly F, Ellis J (2000). Experimental infection of colostrum deprived piglets withporcine circovirus 2 (PCV2) and porcine reproductive and respiratory syn-drome virus (PRRSV) potentiates PCV2 replication. Arch Virol.

[29] Jung K, Kim J, Ha Y (2006). The effects of transplacental porcine circovirus type 2 infection on porcine epidemic diarrhoea virus-induced enteritis in preweaning piglets. VetJ.

[30] Ellis JA, Allan G, Krakowka S (2008). Effect of coinfection with genogroup 1 porcine torque teno virus on porcine circovirus type 2-associated postweaning multisystemic wasting syndrome in gnotobiotic pigs. Am J Vet Res.

[31] Darwich L, Segales J, Mateu E (2004). Pathogenesis of postweaning multisystemic wasting syndrome caused by porcine circovirus 2: an immune riddle. Arch Virol.

[32] Krakowka S, Ellis JA, McNeilly F (2002). Immunologic features of porcine circovirus type 2 infection. Viral Immunol.

[33] Marion K, Matthias R, Matthias E (2008). Reduction of PMWS-associated clinical signs and co-infections by vaccination against PCV2. Vaccine.

[34] Allan GM, McNeilly F, Kennedy S (2000). 2000b. Immunostimulation, PCV-2 and PMWS. *Vet*. Rec.

[35] Rovira A, Balasch M, Segalés J (2002). Experimental inoculation of conventionalpigs with porcine reproductive and respiratory syndrome virus and porcinecircovirus 2. J Virol.

[36] Opriessnig T, Thacker EL, Yu S (2004). Experimental reproduction of postweaning multisystemic wasting syndrome in pigs by dual infection with Mycoplasma hyopneumoniae and porcine circovirus type 2. Vet Pathol.

[37] Hai YW, Zhi XF, Yu ZW (2016). The effects of Mycoplasma hyopneumoniae on porcine circovirus type 2 replication in vitro PK-15 cells. ResVetSci.

[38] Opriessnig T, Madson DM, Roof M (2011). Experimental reproduction of porcine Circovirus type 2 (PCV2)-associated enteritis in pigs infected with PCV2 alone or concurrently with Lawsonia Intracellularis or salmonella typhimurium. J Comp Pathol.

[39] Kollef MH, Eisenberg PR, Ohlendorf MF (1996). The accuracy of elevated concentrations of endotoxin in bronchoalveolar lavage fluid for the rapid diagnosis of gram-negative pneumonia. Am J Respir Crit Care Med.

[40] Pugin J, Auckenthaler R, Delaspre O (1992). Rapid diagnosis of gram negative pneumonia by assay of endotoxin in bronchoalveolar lavage fluid. Thorax.

[41] Tu PY, Tsai PC, Lin YH (2015). Expression profile of Tolllike receptor mRNA in pigs co-infected with porcine reproductive and respiratory syndrome virus and porcine circovirus type 2. Res Vet Sci.

[42] Van RK, Nauwynck H (2000). Proinflammatory cytokines and viral respiratory disease in pigs. Vet Res.

[43] Van GS, Van RK, Pensaert M (2003). Interaction between porcine reproductive-respiratory syndrome virus and bacterial endotoxin in the lungs of pigs: potentiation of cytokine production and respiratory disease. J Clin Microbiol.

[44] Song LQ, Li LF, Deng KB (2011). Porcine reproductive and respiratory syndrome virus and bacterial endotoxin act in synergy to amplify the inflammatory response of infected macrophages. Vet Microbiol.

[45] Van HD, Pang YT, Pei CT (2015). Expression of toll-like receptor signaling-related genes in pigs co-infected with porcine reproductive and respiratory syndrome virus and porcine circovirus type 2.Res. Vet Sci.

[46] Fu S, Xu L, Li S (2016). Baicalin suppresses NLRP3 inflammasome and nuclear factor-kappa B (NF-kappaB) signaling during Haemophilus parasuis infection. Vet Res.

[47] Yu J, Wu J, Zhang Y (2012). Concurrent highly pathogenic porcine reproductive and respiratory syndrome virus infection accelerates Haemophilus parasuis infection in conventional pigs. Vet Microbiol.

[48] Cloward JM, Krause DC (2009). Mycoplasma pneumoniae J-domain protein required for terminal organelle function. Mol Microbiol.

[49] Virginio VG, Gonchoroski T, Paes JA (2014). Immune responses elicited by Mycoplasma hyopneumoniae recombinant antigens and DNA constructs with potential for use in vaccination against porcine enzootic pneumonia. Vaccine.

[50] Yang W, Chong L, Ying F (2012). Transcription analysis on response of porcine alveolar macrophages to *Haemophilus parasuis*. BMC Genomics.

[51] Liu J, Bai J, Zhang L (2013). Hsp70 positively regulates porcine circovirus type 2 replication in vitro. Virology.

[52] Nagy PD, Wang RY, Pogany J (2011). Emerging picture of host chaperone and cyclophilin roles in RNA virus replication. Virology.

